# PREDICTING USE OF ADVANCED DRIVER-ASSISTANCE SYSTEMS IN OLDER DRIVERS THROUGH STRUCTURAL EQUATION MODELING

**DOI:** 10.1093/geroni/igae098.1313

**Published:** 2024-12-31

**Authors:** Abigail Hansen, Kim Kiely, Tuki Attuquayefio, Diane Hosking, Ranmalee Eramudugolla, Kaarin Anstey

**Affiliations:** University of New South Wales, Sydney, New South Wales, Australia; University of Wollongong, Wollongong, New South Wales, Australia; Western Sydney University, Sydney, New South Wales, Australia; National Seniors Australia, Canberra, Australian Capital Territory, Australia; University of New South Wales, Sydney, New South Wales, Australia; University of New South Wales, Sydney, New South Wales, Australia

## Abstract

Older drivers are an at-risk population when on-road. Normal ageing processes, such as declines in visual, sensorimotor and cognitive processes, impact driving skills and contribute to the increasing crash rate. Advanced Driver-Assistance Systems (ADAS) may provide a solution to keep older drivers safe on the road. This study investigated what technology acceptance and trust factors predict use of ADAS between older drivers who have ADAS in their car and a) use it (n=790) or b) do not use it (n=32). We conducted an online survey to 1330 Australian drivers aged 65 years or older (M=72, SD=6.9, 23% women) in partnership with National Seniors Australia. ADAS acceptance was measured by the Partial Automation Acceptance Scale, measured by the latent factors attitudes towards ADAS, attitudes towards technology, perceived risk and trust. Structural equation modeling examined the structural relationships between use of ADAS, the acceptance scale, locus of control and driver self-efficacy. The results indicated positive attitudes towards ADAS had a significant positive direct effect on use (y =.06, S.E =.02, p =.002 (β =.26)) and trust in ADAS had a significant negative direct effect on use (y = -.03, S.E =.01, p =.024 (β = -.15)). The model tested accounted for 93.4% of the variance in use of ADAS. These findings highlight the importance of older drivers to hold positive attitudes and trust in ADAS in order for them to use it, highlighting potential factors that could be targeted in behavioural change interventions seeing to improve usage rates.

